# Block Copolymer/DNA Vaccination Induces a Strong Allergen-Specific Local Response in a Mouse Model of House Dust Mite Asthma

**DOI:** 10.1371/journal.pone.0085976

**Published:** 2014-01-31

**Authors:** Camille Rolland-Debord, David Lair, Tiphaine Roussey-Bihouée, Dorian Hassoun, Justine Evrard, Marie-Aude Cheminant, Julie Chesné, Faouzi Braza, Guillaume Mahay, Vincent Portero, Christine Sagan, Bruno Pitard, Antoine Magnan

**Affiliations:** 1 Unité Mixte de Recherche, Institut National de la Sante et de la Recherche Médicale (U1087), Centre national de la recherche scientifique (6291), Nantes, France; 2 Université de Nantes, l’institut du thorax, Nantes, France; 3 Centre Hospitalier Universitaire, Service d'anatomie et cytologique pathologiques, Nantes, France; Virginia Tech University, United States of America

## Abstract

**Background:**

Allergic asthma is caused by abnormal immunoreactivity against allergens such as house dust mites among which *Dermatophagoides farinae* (Der f) is a common species. Currently, immunotherapy is based on allergen administration, which has variable effect from patient to patient and may cause serious side effects, principally the sustained risk of anaphylaxis. DNA vaccination is a promising approach by triggering a specific immune response with reduced allergenicity.

**Objective:**

The aim of the study is to evaluate the effects of DNA immunization with Der f1 allergen specific DNA on allergic sensitization, inflammation and respiratory function in mice.

**Methods:**

Mice were vaccinated 28 and 7 days before allergen exposure with a Der f1-encoding plasmid formulated with a block copolymer. Asthma was induced by skin sensitization followed by intra-nasal challenges with Der f extract. Total lung, broncho-alveolar lavage (BAL) and spleen cells were analyzed by flow cytometry for their surface antigen and cytokine expression. Splenocytes and lung cell IFN-γ production by CD8+ cells in response to Der f CMH1-restricted peptides was assessed by ELISPOT. IgE, IgG1 and IgG2a were measured in serum by ELISA. Specific bronchial hyperresponsiveness was assessed by direct resistance measurements.

**Results:**

Compared to animals vaccinated with an irrelevant plasmid, pVAX-Der f1 vaccination induced an increase of B cells in BAL, and an elevation of IL-10 and IFN-γ but also of IL-4, IL-13 and IL-17 producing CD4+ lymphocytes in lungs and of IL-4 and IL-5 in spleen. In response to CD8-restricted peptides an increase of IFN-γ was observed among lung cells. IgG2a levels non-specifically increased following block copolymer/DNA vaccination although IgE, IgG1 levels and airways resistances were not impacted.

**Conclusions & Clinical Relevance:**

DNA vaccination using a plasmid coding for Der f1 formulated with the block copolymer 704 induces a specific immune response in the model of asthma used herein.

## Introduction

Asthma is a frequent disease, affecting approximately 300 million people worldwide [1]. Allergic asthma is the most frequent form of the disease, occurring in atopic subjects, and mainly due to indoor allergens such as house dust mites (HDM) and pet fur. In western countries, the most commonly encountered species of HDM responsible for asthma are *Dermatophagoides pteronyssimus* (Der p) and *Dermatophagoides farinae* (Der f).

Allergic asthma results from an allergen-driven Th2-mediated inflammation in which Th2 cells induce plasma cells to produce IgE and activate mast cells and eosinophils to release mediators and cytokines responsible for smooth muscle contraction, epithelial damages and airway narrowing.

Current asthma treatments are based on the control of inflammation by the use of corticosteroids. Inhaled steroids control the vast majority of cases but in some of them oral steroids are necessary to achieve asthma control, providing inacceptable adverse effects. Curative treatments of asthma do not exist, but allergen-specific immunotherapy (SIT) has been proposed for years to induce an immune tolerance towards allergens [2]. SIT consists in administrating increasing doses of allergen through the subcutaneous or the sublingual route. SIT is a long term treatment lasting from 3 to 5 years with at least 50 sub-cutaneous injections over 3 years. It was showed to decrease symptoms and medication requirements in asthma due to HDM and some pollens. SIT was showed to decrease the Th2 commitment of allergen specific CD4+ T cells and induce T regulatory (Treg) and Th1 cells [3]. The usage of SIT is however limited by the requirement of repeated administration, the variable effect from a patient to another, and the sustained risk of anaphylaxis [2].

The use of DNA vaccination is a promising strategy of SIT, with few administrations required to get a strong immune response. Such strategy was proposed before in several studies. Jarman et al showed a decrease in Th2-type cytokines in bronchoalveolar lavage (BAL) after administration of a plasmid containing DNA encoding an immunodominant peptide of Der p1, a major allergen of Der p intramuscularly in asthmatic compared to untreated mice [4]. In addition, the DNA vaccination-induced immune response can be modulated towards a Th1 or Th2 bias when combining adjuvant to the vaccine, as reported by Kim et al who co-administrated DNA of Der p and the Calmette-Guerin bacillus, known for its pro-Th1 immunomodulatory effects [5]. They improved the asthmatic phenotype with increased production of IFN-γ in BAL. However in these studies high quantities of DNA were required, which precludes any application in humans [6]. Formulations associating plasmid DNA in tetrafunctional block copolymer as a vector, allow to safely increase the transfection efficiency of reporter or therapeutic genes in lung, skeletal and cardiac muscle in healthy and animal models compared with results obtained with naked DNA [7]–[10]. It was also showed that the tetrafunctional block copolymer 704 is able to promote low-dose DNA vaccination efficiency [6]. In a previous study, only two injections of a DNA vaccine encoding the Der f1 gene, a major allergen of Der f, formulated with synthetic vector, induced a strong humoral and cellular response with a Th1-bias, reduced airway hyperresponsiveness and Th2 cytokines in BAL from Der f-sensitized mice [11]. This first paper was mainly a proof of concept article in which different timings of DNA vaccination were tested. Inflammation was considered globally and not at the single cell level. In addition pulmonary measurements of lung function were limited to plethysmography assessments. Herein we propose to characterize the immunological response of this vaccine by testing the prophylactic effect of DNA vaccine, analyzing the induced inflammatory response at the single cell level by flow cytometry, and assessing the airways responsiveness by invasive forced oscillation technique, more sensitive than plethysmography.

## Materials and Methods

### Animal Procedures

**Figure 1:**
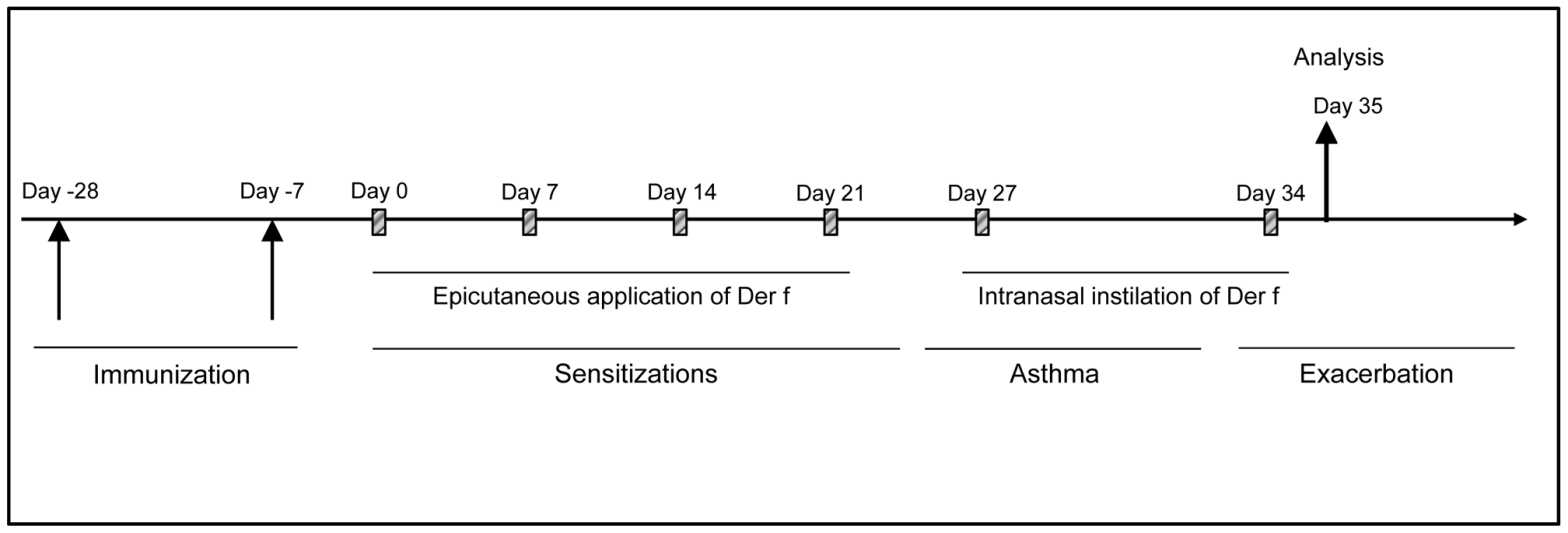
Immunization protocols against Der f1 in asthmatic mice. pVAX-Der f1 or pCMV-βgal plasmid were injected i.m at days −28 and −7. Mice were then epicutaneously sensitized and intranasally challenged with total extract of HDM. Analyses were performed on day 35.

6 weeks Balb/C female mice (Charles River Laboratories, Saint Germain sur l'Arbresle, France) were housed in conventional conditions according to INSERM guidelines and protocol was approved by the Ethics Committee in animal Experimentation of Pays de la Loire (CEEA.2009.22). Two intramuscular (IM) DNA vaccinations were performed at 21 days interval, 28 and 7 days before the onset of sensitization (Day 0). DNA-polymer formulations were injected into both *tibialis anterior* muscles using an Insumed Pic Indolore 30G syringe (Artsana, Grandate, Italy). Mice were anesthetized by isoflurane inhalation, in order to avoid any movement and then ascertain that DNA injections were intra-muscular, and to further prevent pain. In all cases, the injection volume was 50 µl (pVAX-Der f1/704 or pCMV-βgal used as control, 5 µg or 10 µg) per injection site. Seven days after the last vaccination, allergic asthma was induced in Balb/C mice using a total extract of Der f kindly provided by Stallergènes (Antony, France). Mice were sensitized by percutaneous administration of 500 µg of total Der f extract diluted in 20 µl of dimethylsulfoxyde once a week for 4 weeks (Day 0, 7, 14, 21). They were then challenged twice by intranasal inhalation of 250 µg total Der f extract diluted in 40 µl of PBS at day 27 and 34 (Figure 1). During sensitization and challenge, mice were anesthetized by intra peritoneal injection of xylazine and IM injection of ketamine. All analyzes (BAL, lung and spleen cell analyzes, cytokine and immunoglobulin assays, lung function measurements) were performed on samples obtained at day 35, one day after the last challenge. Mice received a sublethal dose of pentobarbital, blood was collected by intra-cardiac puncture serum prepared after centrifugation and stored at −80°C till assays. Bronchoalveolar lavage (BAL) was performed after catheterization of the trachea, with 1 ml of PBS, and lungs and spleen recovered.

Two doses (10 and 5 µg) of pVAX-Der f1/704 were tested. For each dose, experiments were performed in 2 successive and distinct sets of at least 5 mice per group. As the 2 sets of experiments gave comparable results, the data presented are representative of means calculated on combined experiments. Each set comprised 3 groups of mice allergic to Der f, that were compared: a control allergic group free of any intervention, a control allergic group vaccinated by a sham plasmid (pCMV-βgal), and an allergic group vaccinated by pVAX-Der f1/704. A fourth non allergic group was introduced for cell assessment in BAL and serum IgE assays, in order to demonstrate the sensitization induction by Der f.

### Plasmid Preparation and Formulation

The pCMV-βgal ( = pCMV-LacZ) plasmid (Clontech, St Germain en Laye, France) encoding β-galactosidase, and the pVAX-Der f1 plasmid encoding Der f1, under the control of the human cytomegalovirus immediate promoter was used. The pVAX-Der f1 plasmid was obtained by cloning the Der f1 gene sequence into pVAX (Invitrogen, Courtaboeuf, France) with HindIII and Xho1 restriction enzymes. All plasmids were purified using EndoFree plasmid purification columns (Qiagen, Courtaboeuf, France). They were confirmed to be free of endotoxin contamination (endotoxin <0.1 EU/µg plasmid DNA) by the Limulus amoebocyte lysate assay (Lonza, Clermont-Ferrand, France). Then purified plasmids were subjected to quality control by loading 1.5 µg of plasmid DNA with or without restriction enzyme according manufacturer’s protocol (New England Biolab, Ipswich, UK) of an agarose gel 1% containing ethidium bromide (Sigma, Saint-Quentin Fallavier, France). Samples were left to migrate for 30 min at 100 V in Tris–EDTA buffer. The obtained enzymatic restrictions profiles were conform for each plasmid used. The tetrafunctional block copolymer 704 was kindly supplied by In-Cell-Art (Nantes, France) (more Information in Pitard B et al. [8]). Plasmid DNA was formulated immediately prior to IM injection. Two different doses of each plasmid were used (5 and 10 µg).

### Der f1 and βgal Expression Assessment

*Tibialis anterior* muscles were dissected five days after the last vaccine injection, and immediately frozen in RNA stabilizer (Rna Later, Qiagen) at −20°C (n = 3 per group). Muscle tissue was crushed using ULTRA-TURRAX®. RNA was extracted using Rneasy mini kit, (Qiagen) according to the manufacturer’s protocol. To avoid DNA contamination, DNase treatment was performed during 30 min at room temperature with the RNase-Free DNase (Set #79254 QIAGEN) and no residual plasmid was present in the RNA extract as shown by the complete absence of genomic mouse DNA (not shown). Then the RNA was eluted in RNase-free water, measured with a spectrophotometer (Nanodrop) and stored at −20°C. Absence of genomic DNA was confirmed by PCR with mouse genomic DNA as positive control (data not shown).

### Reverse Transcription of RNA

The amounts of RNA obtained were normalized to a concentration of 15 ng/µL (based on the lowest concentration). 16 µl of RNA normalized for all samples at 15 ng/µL were used for RT. RNA was then diluted in a mixture of RNAse-free water and master mix (4 µL), as recommended by the manufacturer protocol (High Capacity RNA- to- cDNA kit, Applied Biosystems). Reverse transcription was performed in four steps: 5 minutes at 25°C, 30 min at 42°C, 5 minutes at 85°C and stored at 4°C. For each condition, PCR was performed with 2 µL of diluted cDNA in a reaction mixture containing 11.2 µL of water, 0.2 µL of Taq DNA polymerase, 2 µL PCR Buffer, 0.6 µL chloride magnesium, 2 µL of deoxynucleotide triphosphate (dNTP), 1 µL of formamide, and 1 µL of primers specific genes of interest, selected using the software “primer 3”. The reaction involves a three-minute denaturation at 94°C, then 35 cycles of amplification (30 seconds of denaturation at 94°C, 30 seconds annealing at 55 Tm for both primer pair, and 45 seconds of elongation at 72°C) followed by a 7 min elongation at 72°C. PCR products were then stored at 4°C. cDNA amplification were controlled by agarosis gel electrophoresis, followed by a revelation using ethidium bromide (BET) under UV light. The absence of genomic mouse DNA in PCR products was verified by specific amplification with mouse specific primers (not shown).

All analyzes described in the sections below were performed at least in duplicate and average results were considered and analyzed.

### Bronchoalveolar Analysis

BAL were obtained and analyzed one day after the last challenge (7 to 9 mice per group). One mL of PBS was instilled intra-tracheally until lungs are inflated uniformly and then slowly aspirated and centrifugated. Total cell number was determined on Kova slide® by optical microscopy. Differential cell counts are determined by flow cytometry with flow cytometer BD LSR II after staining with anti-CD3 APC, anti-CD19 PE-Cy7, FITC anti-F4/80 (eBiosciences, paris, France), anti-CCR3 PE (R&D, Lille, France), anti-PerCP-Cy5.5 Ly6G, anti-CD8 APC-H7 (BD Biosciences, Le Pont-de-Claix,France) and DAPI. Leukocyte populations were identified as neutrophils (Ly6G high), macrophages (F4/80 high DAPI mid (autofluorescence)), eosinophils (CCR3 high), B cells (CD3 négative CD19 high), T cells (CD3 high CD19 négative), and the proportion of CD8+ T cells is determined by the CD8 high population.

### Lung Immune Cells Analysis

Lung immune cells were obtained and analyzed one day after the last challenge (6 to 7 mice per group). Lungs were fragmented and transferred to a conical tube containing digestion solution. Samples were incubated at 37°C under agitation for 1 h. After incubation, cells were dispersed through a 40 µm nylon filter by using a 10 ml syringe. After red blood cells lysis, using a BD FACS Lysing Solution 1X, cells were washed with complete RPMI-1640, passed through a 40 µm nylon filter and resuspended. Total cell counts were determined with Kova slide®. Cytology was determined by flow cytometry as in BAL but on 3.10^6^ cells. Lung cells (8.10^6^ cells/ml) were stimulated for 5 h at 37°C 5% CO2 with phorbol myristate-Acetate (PMA) (50 ng/ml) and ionomycin (500 ng/ml) in presence of monensin (2 ng/ml) for Th17 assessment or brefeldin A (1 ng/ml) for Th1/Th2 and Treg assessment. T cell cytokine production was determined using flow cytometry after cells staining with the following antibodies: fixable viability 450, anti-CD16-CD32, extracellular corresponding marking mix, intracellular labeling mix after fixation and permeabilization (Table 1A).

**Table 1:** Flow cytometry mix for detection of cytokine produced by lung T cells.

MIX	POLYCLONAL STIMULATION	EXTRACELLULAR MARKERS	INTRACELLULAR MARKERS
Treg MIX	.	anti-CD3 PE-Cy7	anti-Il10 FITC
	COMPLETE MEDIUM	anti-CD8 APC-H7	
Th1 MIX	PHORBOLMYRISTATE-ACETATE (PMA).	anti-CD3 APC	anti-IFNγ FITC
		anti-CD8 APC-H7	
Th2 MIX	IONOMYCIN.	anti-CD3 PE-Cy7	anti-Il4 APC
		anti-CD8 APC-H7	anti-Il13 A488
	BREFELDINE A		anti-Il 5 PE
	COMPLETE MEDIUM.	anti-CD3 PE-Cy7	
Th17 MIX	PHORBOLMYRISTATE-ACETATE (PMA).	anti-CD8 APC-H7	anti-Il17 PE
	IONOMYCIN.	anti-CCR6 A488	
	MONENSIN		

### Splenocyte Analysis

Spleens were collected, crushed, homogenized and resuspended in RPMI 1640 containing 10% fetal calf serum, 2 mmol/l L-glutamine, 2-mercaptoethanol, penicillin and streptomycin. Cells (5.10^6^ cells/mL) were cultured in presence of concanavalin A as a positive control (5 mg/ml), purified Der f1 (40 µg/ml) or medium alone for 72 h (n = 2–7 mice per group). Cytokines were assayed with bead-based Luminex® technology using the kit Pro Mouse Cytokine 23-plex designed for assessment of IL-4, Il-5, IL-10, IL-13, IL-17 and IFN-γ (Bio-Rad Laboratories, Munich, Germany). Assays were performed according to the manufacturer's specifications. Data analysis was performed using the Bio-Plex Manager Software version 4.0.

### ELISPOT Assay

As a paradigm read out of the cellular response to vaccine formulation, Der f1-specific CD8+ response was tested by Class I-restricted IFNγ secretion determined by ELISPOT (Abcys, Paris, France). Sixteen 8-mer Der f1-derived peptides predicted to bind with H-2Kb were selected based on the binding score, as calculated by the BIMAS and Net MHC software (Table S1) and were used as representative Der f1 epitopes. The negative control was the KRWIILGLNK peptide (HIV gag 263–272). Splenocytes were resuspended in complete medium (RPMI 1640 supplemented with 10% fetal calf serum, 2 mmol/l L-glutamine, penicillin and streptomycin, all from Invitrogen, Paisley, UK), and then distributed in triplicate at 5.10^5^ cells/well in a 96 wells plate. Cells were incubated overnight at 37°C in 5% CO_2_ in the presence of peptides (4 µg/ml). Lung cells were isolated as previously described and were cultured (1.10^5^ cells/well) and stimulated in the same conditions as the spleen cells. Spot-forming colonies (SFC) were detected according to the manufacturer’s protocol, counted automatically on an AID ELISPOT Reader (Autoimmun Diagnostika, Strassberg, Germany). The nonspecific signal was obtained by averaging the negative control values, thereby eliminating any control variation, and after subtracting the mean value of the sample. Results are expressed as SFC/10^6^ splenocytes (n = 2 to 6 mice per group) or lung cells (n = 3 per group).

### Immunoglobulins Assays

Blood was removed by cardiac puncture and centrifugated to collect serum. Sera were stored at −80°C. Total IgE, IgG1, IgG2a were assessed by ELISA according to manufacturers’ instructions (Bethyl laboratories, Montgomery, TX, USA) (n = 6 to 7 mice per group). Briefly, plates were coated with 100 µL of diluted coating antibody solution. After 1 hour at room temperature, plates were washed and incubated 30 min with PBS/BSA 1%. Detection antibody coupled to HRP (horseradish peroxidase) was then transferred in each well. After one hour TMB substrate was added in the dark at room temperature for 15 min. Elisa Stop solution was added and absorbance measured at 450 nm with VictorTMX3 (PerkinElmer, Courtaboeuf, France). Concentrations of immunoglobulins are calculated from the standard curve taking into account the corresponding dilution factor. In order to have a negative control of sensitization an additional group of mice was added in these experiments. These mice completed the whole protocol except exposure to Der f extract that was replaced by DMSO alone for skin sensitization and PBS for challenges.

### Measurement of Airway Responsiveness

Respiratory function was analyzed with a Flexivent® (SCIREQ) and the forced oscillation method (6 mice for unvaccinated group and 7 to 9 mice for control and Der f vaccinated group). Each mouse was anesthetized with a mix of ketamine/xylasine (0.05 mg/kg), intubated, ventilated and curarized for regular ventilation. Survival of the animal was monitored by electrocardiogram.

To measure respiratory mechanics, the *flexiVent* briefly pauses mechanical ventilation and executes a measurement maneuver during which a predefined pressure or volume waveform is applied to the mice's airway opening. Throughout the maneuver, pressure and volume data are recorded. In order to overcome the heterogeneity, parameters collected were normalized to the 0 mg/ml dose. Analyzes (airway resistance, compliance and lung elastance) were performed after nebulisation of increasing doses of methacholine (0, 5, 10, 15 and 20 mg/ml).

### Statistical Analysis

Statistical analysis was conducted using Graph Prism® software. All data are expressed as mean ± standard deviation. Statistical analysis was performed using Mann-Whitney Test. A p value <0.05 was considered as significant.

## Results

### pVAX-Der f1 Vaccination Induces Der f1 Gene Expression

**Figure 2:**
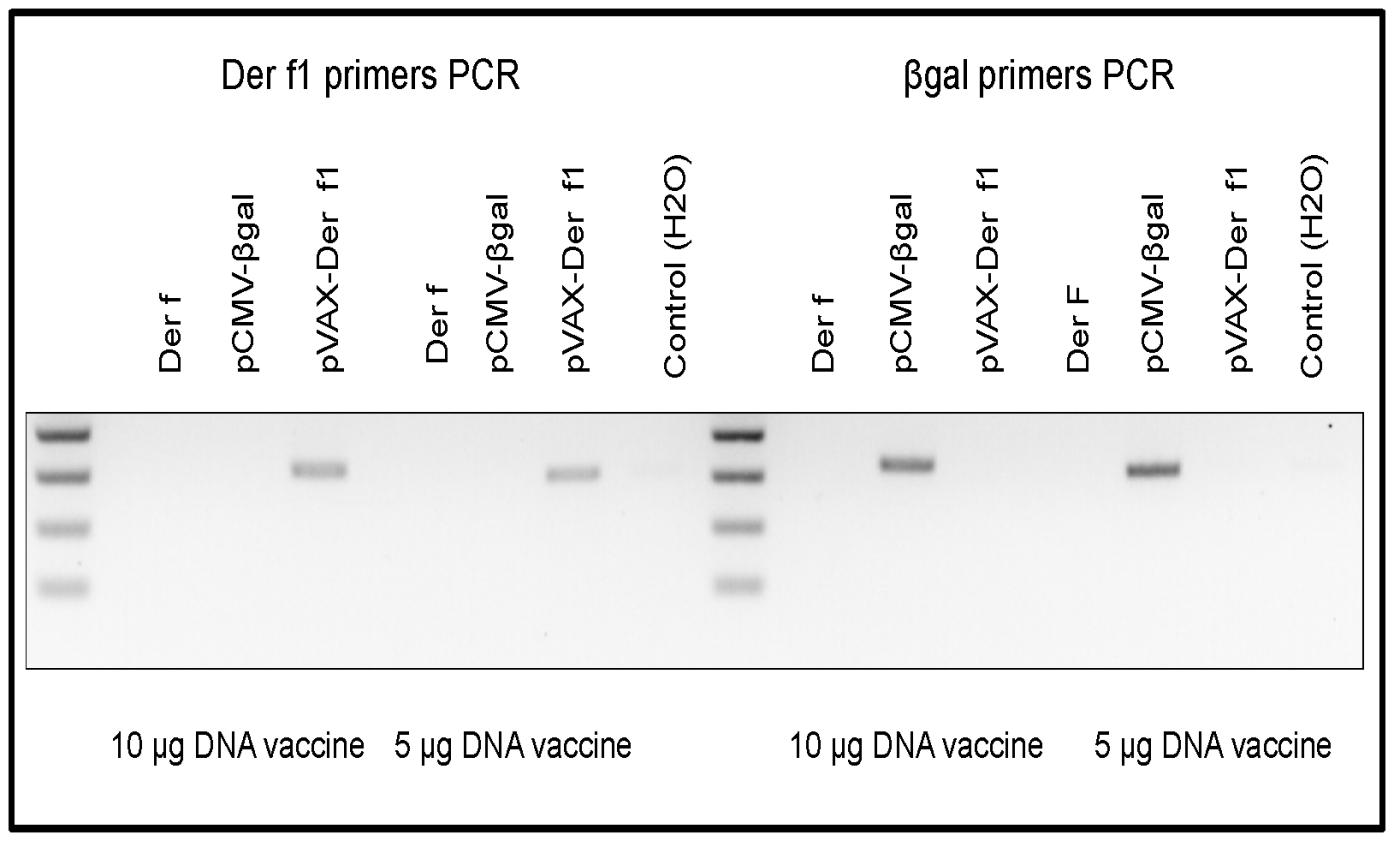
Der f1 and βgal expression assessment. Five days after the last vaccine injection, Der f1 and βgal gene expression were verified by RT-PCR on skeletal muscle RNA using specific primers (n = 3 per group). This experience was done for both DNA vaccination conditions (10 µg and 5 µg of plasmid preparation).

Beilvert et al previously described the Der f1 expression in muscle tissue 5 days after the last DNA injection at the protein level [11]. To verify the Der f1 production by muscle cells, we performed a qualitative detection of Der f1 mRNA in *Tibialis anterior* muscle. Expression of βgal and Der f genes was verified 5 days after vaccination by RT-PCR (Figure 2). Myocytes from mice vaccinated either by 10 or 5 µg DNA per injection expressed specifically Der f1 or βgal mRNA.

### pVAX-Der f1 Vaccination Modulates the Lung B cell Infiltration

Effects of DNA vaccination on Der f-induced pulmonary inflammation was assessed by comparing results obtained in vaccinated mice to these obtained in unvaccinated mice and in mice injected with an irrelevant plasmid coding for the βgal gene.

**Figure 3:**
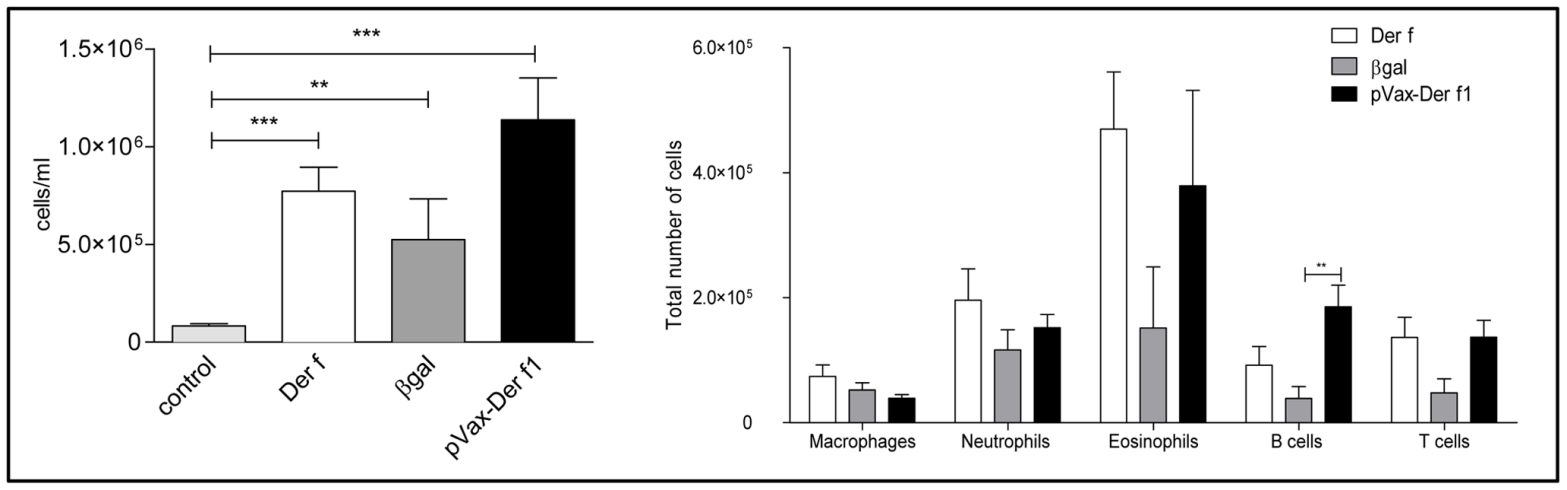
Effect of immunization protocol on BAL inflammation of non asthmatic non vaccinated mice (light grey bar, n = 8), Der f mice (open bar, n = 9), pCMV-βgal (grey bar, n = 7) and pVAX-Der f1 mice (black bar, n = 7) when using 10 µg of DNA. The total number of cells was determined by cell count on Kova slides. The cellular composition was established by flow cytometry. Results are expressed as absolute number of cells, as the mean and standard deviation for each group. **p<0,01 and *** p<0,001 by Mann Whitney test.

First, total BAL cells were analyzed by flow cytometry (Figure 3). In control allergic mice intra-nasal Der f challenges elicited a high cellular influx in BAL mainly due to an increased number of eosinophils. Treatment with 10 µg of pVAX-Der f1 caused an increase of B cells that was significant compared to mice injected with the control plasmid (p = 0.007). No significant modulation of other cell types was observed. Similar results were obtained with 5 µg DNA (data not shown).

Then, to check whether DNA vaccination effects were induced at the tissue level, lungs were digested and inflammatory cell populations were assessed by flow cytometry, one day after the last challenge. Whatever the dose of DNA injected, there was no significant difference in lung inflammation or representation of different types of sub-populations (macrophages, eosinophils, neutrophils, and lymphocytes) in the Der f1-treated mice compared to the two other groups (data not shown).

### pVAX-Der f1 Vaccination Modulates T cell Activation in Lungs

**Figure 4:**
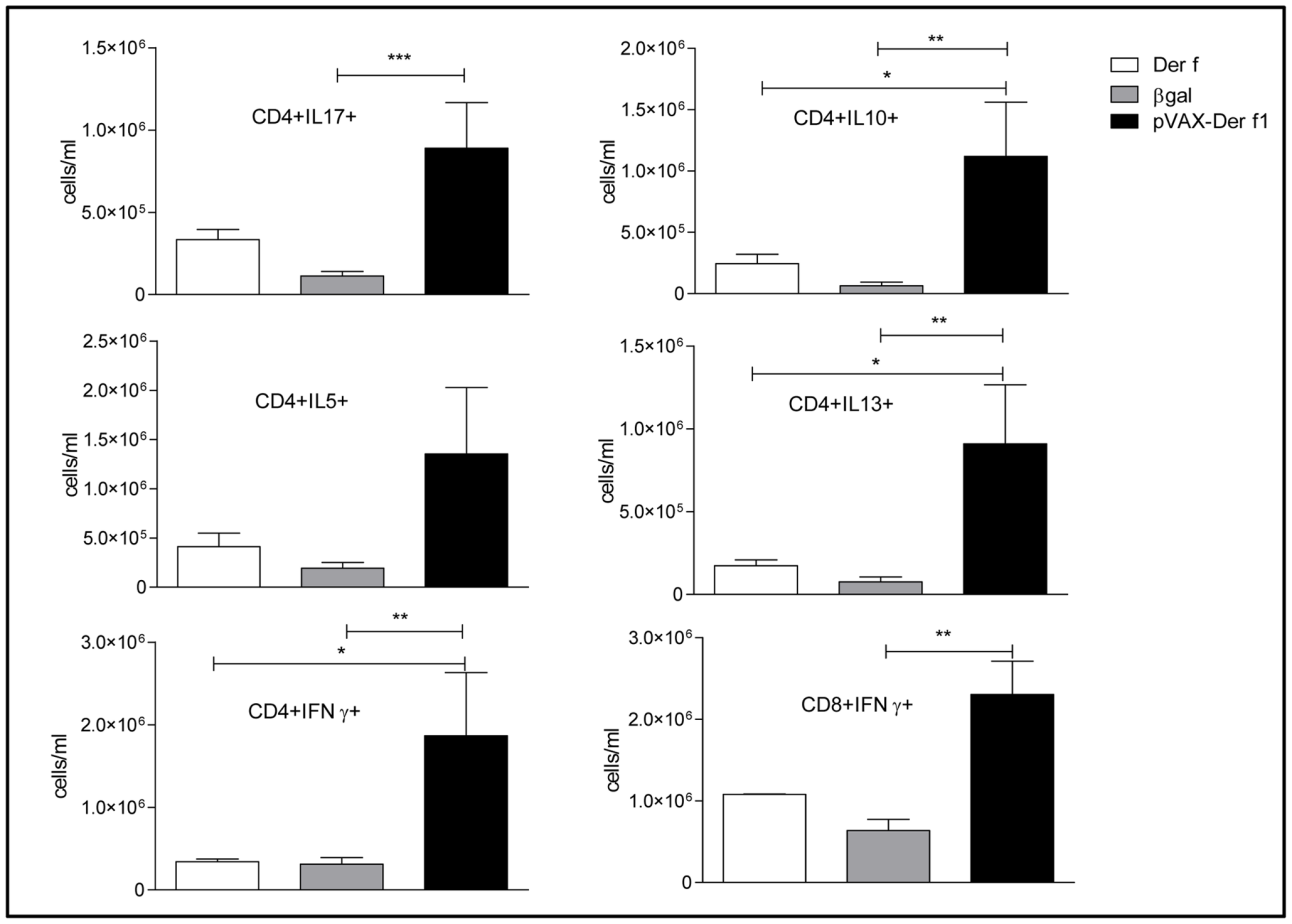
Effect of immunization protocol on T cells cytokines secretion of Der f mice (open bar, n = 6), pCMV-βgal (grey bar, n = 7) and pVAX-Der f1 (black bar, n = 7) when using 10 µg of DNA. One day after the last airway allergen challenge, mice were sacrificed and lung cells were cultured. The concentration of IL-4, IL-5, IL-13, IFN-γ, IL-10 and IL-17 were measured by flow cytometry. Results are expressed as the mean and standard deviation for each group. *p<0,05, ** p<0,01 and *** p<0,001 by Mann Whitney test.

To further characterize the T cell response elicited by DNA vaccination we then quantified the lung cytokine production by CD4+ T cells at the single cell level by flow cytometry (Figure 4). Th1 CD4+ cells from pVAX-Der f1 vaccinated mice, identified as CD4+IFN-γ producing T cells, were increased after allergen challenge, compared to pCMV-βgal immunized mice (p = 0.0041) and unvaccinated mice (p = 0.0177). The IFN-γ production by CD8+ T cells was also increased in pVAX-Der f1 treated mice compared to βgal treated mice (p = 0.0012) but not unvaccinated mice. Treg cells defined as IL-10 producing CD4+ T cells increased in response to pVAX-Der f1 vaccination by comparison to both control groups (p = 0.0021 vs pCMV-βgal and p = 0.0140 vs unvaccinated asthmatic mice). However the treatment also induced an increase of Th2 cells, characterized by a significant increase of IL-13-producing CD4+ cells (p = 0.0012 vs pCMV-βgal and p = 0.0101 vs controls). No increase of IL-5+CD4+ cells was observed (p = 0.1649). Th17 cells also increased, but vs βgal vaccinated control mice only (p = 0.0006).

Vaccination with 5 µg of DNA had no effect on T cell cytokine production (data not shown).

### pVAX-Der f1 Vaccination Induces a Specific Peripheral Immune Response

To assess whether DNA vaccine affected the systemic adaptive immune response splenocytes were stimulated ex vivo with purified Der f1 and T cell derived cytokines were assayed in culture supernatants (Table 2). Der f1 stimulation of splenocytes from pVAX-Der f1 treated mice induced a significant increase in IL-4 and IL-5 production. The increase of IFN-γ, IL-10, IL-13 and IL-17 production did not reach statistical significance.

**Table 2:** Cytokine concentrations (pg/ml) in vaccinated mice spleen cell culture supernatants after stimulation by medium of Der f1.

	medium			Der f1		
	Der f	pCMV-βgal	pVAX-Der f1	Der f	pCMV-βgal	pVAX-Der f1
**IL4**	10.5±7.9	3.6±3.9	7.3±7.8	79.6±31	59.1±1.2	164.9±72.7 *
**Ifn-γ**	6.3±4.1	3.1±0.98	2.7±1.96	446±248.8	679±494.5	2174.7±1520.6
**IL10**	10.8±5.4	6.2±4.4	6.9±5.7	76.5±41.9	65.3±12.3	390.5±363.2
**IL5**	1.8±1	0.8±0.1	1.35±1.06	67.1±46.5	49.5±40.5	843.5±626.2 * **^$^**
**IL13**	27±38.2	2.7±3.8	14.28±20.16	381.8±219.1	332.1±36.6	2831.6±3114.8
**IL17**	2.6±1.4	1.4±0.3	1.7±0.9	456.3±687	1687.4±513	2257.8±2531.7

* p<0,05 between pCMV-βgal and pVAX-Der f1 and ^$^ p<0,05 between Der f and pVAX-Der f1.

**Figure 5:**
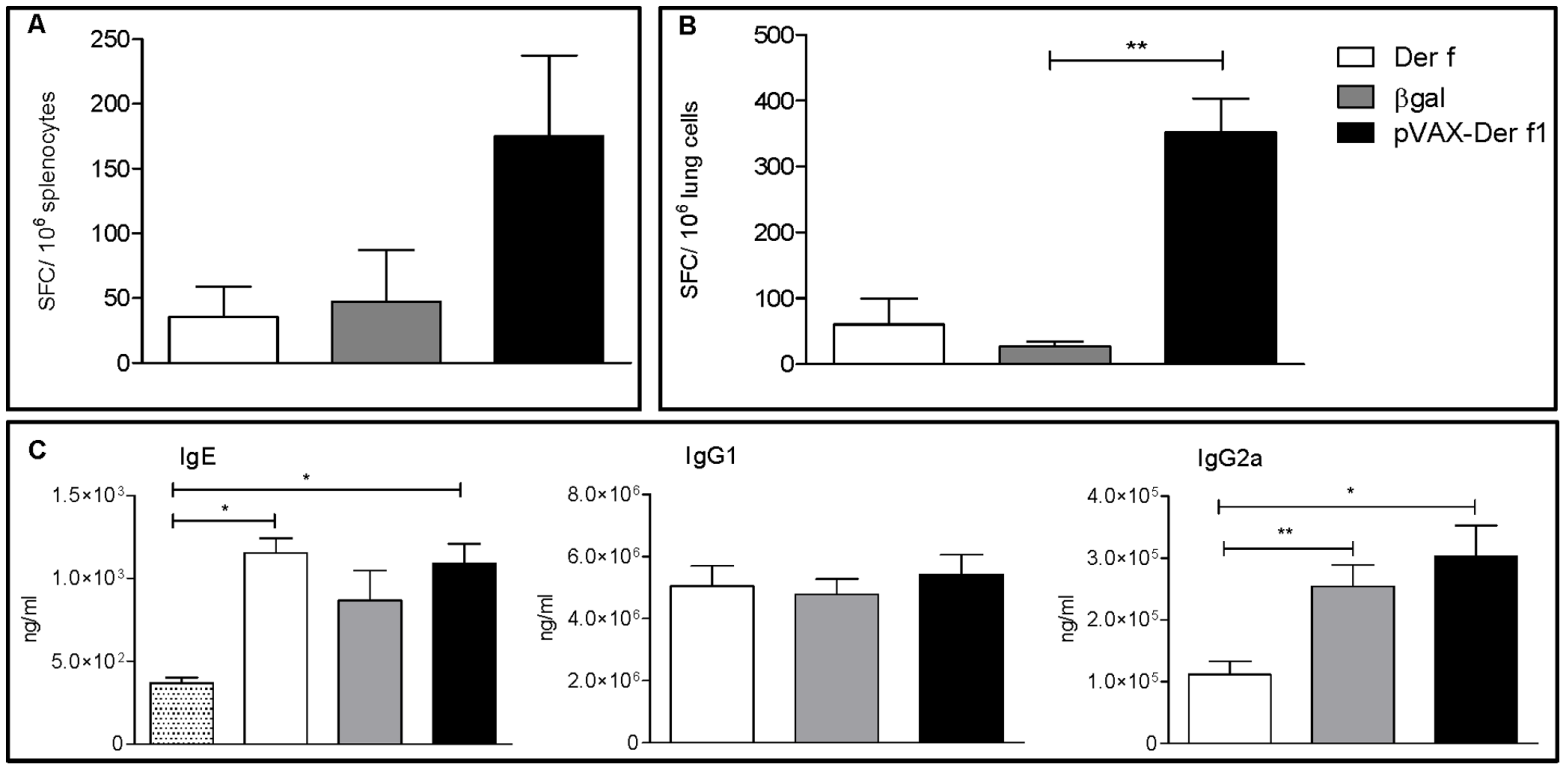
Effect of immunization protocol on the immune response of asthmatic mice. A. Splenocytes were stimulated overnight with a pool of Der f1 immunodominant peptides. The number of IFN-γ SFCs was determined. Der f (n = 2), pCMV-βgal (n = 5), pVAX-Der f1 (n = 6). B. Lung cells were stimulated overnight with a pool of Der f1 immunodominant peptides. n = 3 mice per group. The number of IFNγ spot forming colonies (SFCs) was determined. C. Humoral response was measured in sera one day after the last challenge in Der f (n = 6), pCMV-βgal (n = 7) and pVAX-Der f1 (n = 7) mice and additionally in control mice for IgE. Results are expressed as IgE, IgG1, IgG2a antibody titer. The mean number and standard deviation are shown for each group. *p<0,05 and ** p<0,01 by Mann Whitney test.

To further characterize the specific cellular immune response after DNA vaccination, splenocytes and lung cells were stimulated *in vitro* with a cocktail of CD8 immunodominant Der f1 peptides. There was an increase of IFN-γ producing lung T cells in Der f1 vaccinated mice compared to pCMV-βgal vaccinated mice (p = 0.0043). At the splenocyte level, the increase was not significant (Figure 5 A & B). This experiment demonstrated that Der f1 specific CD8+ cells producing IFN-γ were induced in lungs from pVAX-Der f1 immunized mice.

IgE and IgG1 induced during a Th2 response and IgG2a induced during a Th1 response were measured in the serum of mice immunized with Der f1 or βgal or in unvaccinated mice. In addition, to demonstrate that mice were well sensitized by Der f extract, IgE were assayed in sera from non allergic mice. Serum immunoglobulin quantification was done by ELISA (Figure 5C). Total IgG2a levels in vaccinated mice were significantly increased in sera (p = 0,014 for pVAX-Der f1 and p = 0,0047 for pCMV-βgal) compared to unvaccinated mice, whereas both total IgE and IgG1 were similar in all groups. IgE levels from asthmatic mice, either immunized with Der f1 or unvaccinated were significantly increased compared to control non sensitized non vaccinated mice. Compared to mice vaccinated with a control plasmid, the difference was not significant.

### pVAX-Der f1 Vaccination does not Affect Der f-induced Airway Responsiveness

**Figure 6:**
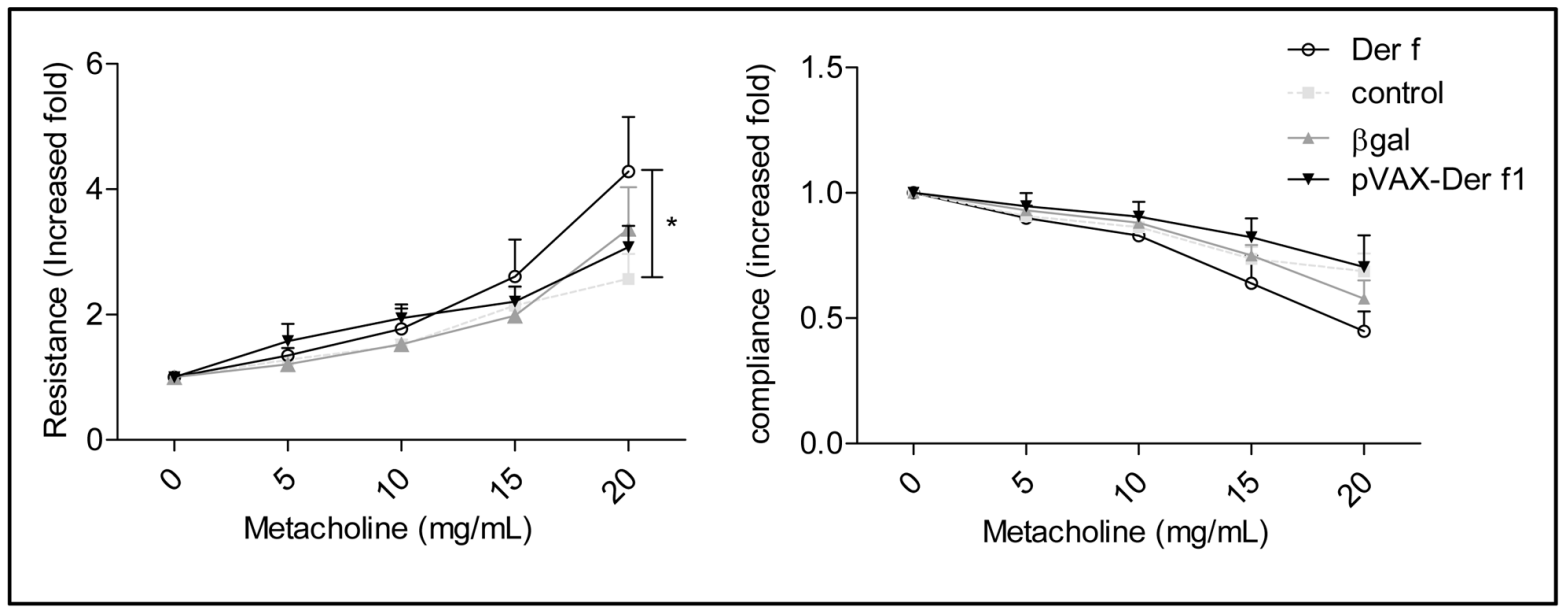
Effect of prophylactic immunization protocol with 10 µg of Der f1 DNA on respiratory function. Airway resistance and Compliance was measured at day 35 using Flexivent with instillation of 5 to 20/ml methacholine in non asthmatic non vaccinated mice (n = 6, 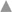) Der f (n = 7, O ), pCMV-βgal mice (n = 9,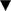) and pVAX-Der f1 (n = 9,▾) mice. Results are expressed in increased fold, as a mean for each group ± standard deviation. *p<0,05 by Mann Whitney test.

The consequences of pVAX-Der f1 vaccination-induced immune response on airways hyperresponsiveness were then assessed. Tracheotomized mice were ventilated by Flexivent® (scireq, Montreal, Canada) 24 hours after the last intranasal challenge (Figure 6), and airways resistances directly measured after instillation of increasing doses of methacholine. Der f extracts induced airways hyperresponsiveness in response to methacholine, which was conserved in Der f1 vaccinated mice compared to both control groups. These results demonstrated that pVAX-Der f1 vaccination effects were not sufficient in this model to reverse the asthmatic phenotype in HDM allergic mice.

## Discussion

The aim of this study was to investigate the allergen specific DNA vaccination-induced effects in a murine model of allergic asthma driven by sensitization to *Dermatophagoides farinae*. Our results demonstrate that pVAX-Der f1 vaccine primes the immune system to build a T cell activation upon allergen challenge of sensitized mice, with an increase of not only the expected protective Th1 and Treg cells but also Th2 and Th17 cells. In a previous work designed to compare the immune response induced by pVAX-Der f1 formulated with various copolymers [11], we showed that vaccination with the same compound promoted a shift from a Th2 to a Th1 response, materialized by an increase of IFN-γ, a decrease of IL-5 by BAL cells, and an increase of the IgG2a/IgG1 ratio. In addition a decrease of BAL inflammatory cells was found in vaccinated mice, as well as a non significant decrease of airways hyperresponsiveness measured by plethysmography. To go further into the characterization of the immune allergic and functional responses induced with the best protocol and the best copolymer formulation, and also compare 2 doses of plasmid, the present study used methods more sensitive and more specific: the pulmonary inflammation and the T cell cytokine production were assessed at the single cell level by flow cytometry; airways hyperresponsiveness was assessed by forced oscillations, which provides resistance and compliance directly measured in the lower airways. All experiments were repeated at least in duplicate and gave comparable results, so that data presented are mean results combining all experiments.

Our results show that pVAX-Der f1 vaccine induces a strong immune response specific of Der f1, as assessed by the effect of the intra-nasal challenge in vaccinated mice on B cell infiltration and T cell cytokine production, the IFN-γ production by CD8+ cells stimulated by Der f1-derived peptides and the production of specific immunoglobulins in vaccinated asthmatic mice. This clear-cut specific response does not induce any exacerbation of asthma despite the induction of Th2 and Th17 cytokines. Indeed there is no increase of inflammatory cells in BAL compared to non vaccinated mice at the exception of B cells, and no increase of bronchial hyperrresponsiveness. The vaccination process can therefore be considered as well tolerated, whatever the dose of DNA used. It is noteworthy that albeit Der f1 vaccination induced a clear specific response, vaccination with a control irrelevant plasmid non-significantly downregulated some inflammatory parameters and induced IgG2a production. This effect could be due to a non specific effect of the plasmid/copolymer block that should be studied further. Indeed this effect could be beneficial in reducing inflammation. It could be related to pathogen-associated molecular patterns that would non-specifically stimulate innate immunity. Accordingly IgG2a levels, related to Th1 activation, were increased in the same proportion in Der f-vaccinated mice and mice vaccinated by the irrelevant compound. The consequence of this effect for the present study could explain in part that some significant differences could be observed in Der f-vaccinated mice by comparison to mice vaccinated with the βgal compound but not to asthmatic non vaccinated mice. This strengthens the effect of the specific Der f1 DNA sequence, but attenuates the global effect of DNA vaccination. The absence of difference with control asthmatic mice was also explained by a lower number of mice in this group that we essentially considered as a positive control for asthma induction, the real control of vaccination being the pCMV-βgal plasmid.

Although it is not protective, an increase of Th1 and Treg cells is observed, materialized by the increase in IgG2a/IgG1 ratio and IFN-γ, and of IL-10 producing CD4+ T cells when injecting a dose of 10 µg of DNA. Such Th1/Treg profile is considered as the substratum of the protection conferred against allergens by SIT. Indeed early studies on the mechanisms of SIT have showed a Th1 bias induced by this treatment [12], [13] whereas more recent publications have highlighted the role of Treg cells in this process [14], [15]. Also, the induction of a specific Th1/Treg response was showed in other DNA vaccination-based experiments. For instance, an established Th2 allergic inflammation can be attenuated through an IL-10-dependent mechanism with DNA encoding the chaperone protein Hsp65 [16].

The DNA vaccine-induced immune response is likely dependent on the local production of cytokines such as IL-12, secreted through cross-presentation of the allergen by transfected muscle cells to professional antigen-presenting cells [17] inducing the specific IgG2a production by plasma cells, counterbalancing in turn the effect of IgE. In this view Peng *et al*. have demonstrated that the beneficial effect of an allergen DNA vaccine depended on the IgE regulation in a T cell dependent manner [18].

The efficacy of pVAX–Der f1 vaccine could be improved by optimizing the immunization protocol using the vaccine consisting of the block copolymer and the plasmid encoding Der f1. The vaccination vector used belongs to a new class of synthetic vector, a tetrafunctional block copolymer, able to safely increase the gene transfection efficiency in skeletal muscle compared with results achieved using naked DNA [11]. The tetrafunctional block copolymer 704 used here is able to promote low-dose DNA vaccination efficiency without any detectable deleterious effect and was chosen for its clear efficiency in inducing a specific response [11]. Other copolymers have also been described as able to direct a more Th1/Treg biased response that could be explored in the protection against asthma in the present mouse model [19].

In addition, the doses of DNA injected were chosen to elicit an optimized immune response. Lower doses could be tested that could privilege the induction of a protective effect. Lastly the vaccination protocol was set up according to preliminary results indicating an optimal expression of the protein at 3 weeks after injection. This delayed peak of Der f1 expression prompted us to vaccine mice before sensitization to get a maximal Der f1 expression at time of asthma induction. Such preventive protocol could achieve HDM prevention in at risk children. However other schedules of vaccination inducing a lower expression of Der f1 at time of asthma triggering should also be tested.

It is noteworthy that the model of HDM-induced asthma used herein is closely related to the reality of asthma with a transcutaneous allergen sensitization, and a mixed Th1/Th17 inflammation induced by Der f intra-nasal challenges, responsible for eosinophil and neutrophil pulmonary infiltrate. This model could be less sensitive to allergen specific immunomodulation compared to a more Th2-commited model. Indeed in clinical practice SIT is more efficient in pure Th2-driven eosinophilic allergic asthma than in more severe forms of asthma frequently associated to a neutrophilic contingent of inflammatory cells. This restriction would be a serious obstacle to the development of DNA vaccination in clinics. In literature, most DNA vaccination studies were performed with a preventive strategy. However, patterns of vaccination differ among studies so that comparison with our protocol is difficult [18]–[20]. Further studies with tetrafunctional block copolymer-based vaccination will have to be performed with optimized procedures to induce a consistent effect not only on immunity but also airways hyperresponsiveness.

In conclusion, DNA vaccination using a plasmid coding for Der f1 formulated with the tetrafunctional block copolymer 704 induces a specific humoral and cellular response against Der f1 in the present mouse model of asthma induced by Der f by comparison to mice vaccinated with an irrelevant plasmid.

## Supporting Information

Table S1 **Prediction analysis of Der f1-specific, CD8-restricted immunodominant peptides.** The tables display sequences of the 16 selected 8-mer peptides susceptible to bind with H-2Kb with their relative ranks (when higher than 12^th^) in BIMAS (www-bimas.cit.nih.gov/molbio/) and NetMHC (http://www.cbs.dtu.dk/services/NetMHC/) algorithms.
(DOCX)
