# Supplementary material for: Block Copolymer/DNA Vaccination Induces a Strong Allergen-Specific Local Response in a Mouse Model of House Dust Mite Asthma
Source: PLoS One. 2014 Jan 31;9(1):e85976. doi: 10.1371/journal.pone.0085976 (PMC3908923; doi:10.1371/journal.pone.0085976)
Supplement: Table S1 — Prediction analysis of Der f1-specific, CD8-restricted immunodominant peptides. The tables display sequences of the 16 selected 8-mer peptides susceptible to bind with H-2Kb with their relative ranks (when higher than 12th) in BIMAS (www-bimas.cit.nih.gov/molbio/) and NetMHC (http://www.cbs.dtu.dk/services/NetMHC/) algorithms. (DOCX) [file pone.0085976.s001.docx]

Block copolymer/DNA vaccination induces an allergen-specific local response in a mouse model of house dust mite asthma

**Supporting Information**

|  | | Rank in algorithm | |
| --- | --- | --- | --- |
| *8-mer Peptides* | *Sequence* | BIMAS | Net MHC |
| *Der f1_2* | *FVLAIASL* | - | 5 |
| *Der f1_15* | *VYARPASI* | - | 10 |
| *Der f1_32* | *FNKNYATV* | - | 7 |
| *Der f1_82* | *SAEAFEQL* | 9 | 8 |
| *Der f1_131* | *SCWAFSGV* | - | 1 |
| *Der f1_147* | *LAYRNTSL* | 4 | 2 |
| *Der f1_190* | *RSYPYVAR* | - | 4 |
| *Der f1_206* | *NSQHYGIS* | 6 | - |
| *Der f1_211* | *GISNYCQI* | 3 | - |
| *Der f1_264* | *YQPNYHAV* | 5 | 9 |
| *Der f1_280* | *QGVDYWIV* | 10 | - |
| *Der f1_298* | *SGYGYFQA* | 2 | 6 |
| *Der f1_302* | *YFQAGNNL* | 7 | - |
| *Der f1_310* | *MIEQYPYV* | - | 3 |
| *Der f1_313* | *EQYPYVVI* | 1 | - |
| *Der f1_314* | *QYPYVVIM* | 8 | - |

**Table S1.** Prediction analysis of Der f1-specific, CD8-restricted immunodominant peptides. The tables display sequences of the 16 selected 8-mer peptides susceptible to bind with H-2Kb with their relative ranks (when higher than 12^th^) in BIMAS (www-bimas.cit.nih.gov/molbio/) and NetMHC (http://www.cbs.dtu.dk/services/NetMHC/) algorithms.
